# Involvement in Mental Health and Substance Abuse Work: Conceptions of Service Users

**DOI:** 10.1155/2011/672474

**Published:** 2011-07-26

**Authors:** Minna Laitila, Merja Nikkonen, Anna-Maija Pietilä

**Affiliations:** ^1^Department of Nursing Science, Faculty of Health Sciences, University of Eastern Finland and The Hospital District of South Ostrobothnia, P.O. Box 1627, 70211 Kuopio, Finland; ^2^Department of Nursing Science, Faculty of Health Sciences, University of Eastern Finland, 70211 Kuopio, Finland; ^3^Department of Nursing Science, Faculty of Health Sciences, University of Eastern Finland and Social and Health Care Services, 70211 Kuopio, Finland

## Abstract

Service user involvement (SUI) is a principal and a guideline in social and health care and also in mental health and substance abuse work. In practice, however, there are indicators of SUI remaining rhetoric rather than reality. The purpose of this study was to analyse and describe service users' conceptions of SUI in mental health and substance abuse work. The following study question was addressed: what are service users' conceptions of service user involvement in mental health and substance abuse work? In total, 27 users of services participated in the study, and the data was gathered by means of interviews. A phenomenographic approach was applied in order to explore the qualitative variations in participants' conceptions of SUI. As a result of the data analysis, four main categories of description representing service users' conceptions of service user involvement were formed: service users have the best expertise, opinions are not heard, systems make the rules, and courage and readiness to participate. In mental health and substance abuse work, SUI is still insufficiently achieved and there are obstacles to be taken into consideration. Nurses are in a key position to promote and encourage service user involvement.

## 1. Introduction

Service user involvement (SUI) is emphasized in many strategies, plans, and declarations. It is a recognized value in social work and in health care, yet in practice, SUI is not always achieved [1–3]. There is a dissonance between the philosophies of SUI and the existence of these philosophies in the reality of mental health nursing practice [4]. SUI is a difficult and complex concept to define, and it is often used as a synonym for participation [5]. On the other hand, a distinction can be made between these two concepts. Service user involvement entails preconditions of the service user's impact on services in some way while user participation can mean users merely taking part in an activity or acting as an informant [6]. 

There are two main models behind SUI. Consumerism or the ethos of markets sees service users as customers, consumers or stakeholders whose views need to be taken into account [7]. Consumerism can be described as a “top-down” [8] or “means to an end” [9] approach where service user involvement serves the interests of the organizations, service systems, and markets. In contrast, the democratic, or empowerment model is concerned with service users having a voice in services, civil rights, and equal opportunities [7]. The empowerment model is more about the “bottom-up” interests of service users themselves [8], and involvement is seen and valued as an “end in itself” [9]. Rutter et al. [10] state that user involvement has the ability to improve the quality of care, but equally important is the potential effect it has on the service users' personal and collective identity, sense of self-worth, and civil rights.

In Finland, SUI is exercised mainly through local democratic mechanisms, elections, and patient organizations. There are also acts covering the rights and status of patients [11], social service clients [12], and complaints systems [13]. The national plan for mental health and substance abuse work [14] was introduced in 2009, and it outlines common national objectives for mental health and substance abuse work. The plan emphasizes that the client's status must be reinforced; user experts and peers should be included in the planning, implementation, and evaluation of mental health and substance abuse work. 

As Goodwin and Happell [15] point out, published research about service user involvement and participation is limited, and there is a lack of research reflecting the views and opinions of service users themselves. The purpose of this study was to describe service user involvement in mental health and substance abuse work from the viewpoint of clients. The following study question was addressed: what are service users' conceptions of SUI in mental health and substance abuse work?

## 2. Materials and Methods

This study applied a phenomenographic approach. This approach was first developed in the field of education to describe qualitatively different ways in which people conceive learning [16]. Phenomenography is a research approach which aims to study and describe different conceptions people have of various phenomena in the world around them [17, 18]. 

In the phenomenographic approach, conceptions are understood as a relationship between an individual and the surrounding world [19]. So the meaning of conception is deeper and wider than in everyday language [20]. Conception has two intertwined aspects: the referential aspect, which denotes the meaning of the object conceptualized, and the structural aspect, which shows the specific combination of features that have been discerned and focused on [21]. The phenomenographic approach makes a distinction between how something is, and how it is perceived to be. These are called first-order and second-order perspectives. The first-order perspective is directed towards the phenomenon as such and it is interested in facts, whereas the second-order perspective is about a person's conceptions of that phenomenon. The second-order perspective is studied in phenomenography [18, 22, 23].

When applying a phenomenographic approach, the second-order perspective is adapted throughout the research project. When study questions are formulated, data is gathered and the analysis is done. At every stage, the researcher has to reflect and be conscience of her or his own conceptions of the phenomenon of interest. The researcher has to be open to the participants' conceptions [22]. *“It means taking the place of the respondent, trying to see the phenomenon and the situation through her eyes, and living her experience vicariously”* (page 121). However, it is impossible to approach and analyse empirical data totally without preconceived ideas. An empirical study is guided by a specific research interest, and a researcher must be acquainted with previous theory in order to be able to pose relevant questions and to interpret and analyse the data [24]. Theoretical knowledge makes a researcher a valid research instrument [25].

**Figure 1:**
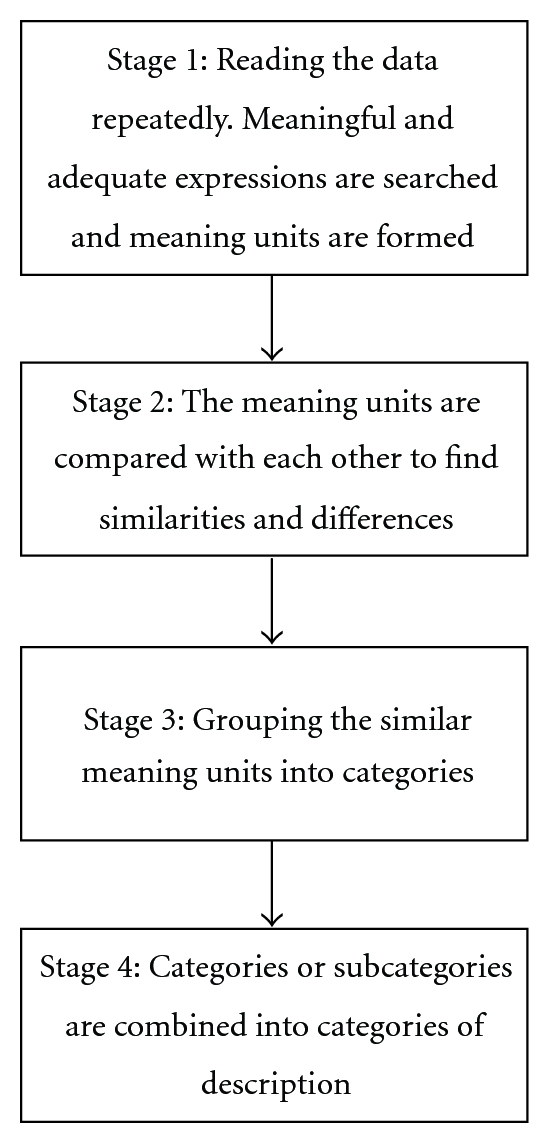
Phenomenographic analysis.

The phenomenographic analysis is not very structured, and it is always based on empirical data [19]. The analysis usually contains different stages [20]. The first stage involves reading the data repeatedly in order to form an overall picture of participants' conceptions. Meaningful and adequate expressions are searched for from the data, and meaning units are formed. In the second stage of the analysis, the meaning units are compared with each other to find similarities and differences. The third stage concerns grouping the similar meaning units into categories. In the fourth stage, categories or subcategories are combined into categories of description. These categories of description represent the results of the study (Figure 1).

### 2.1. Ethical Considerations

The study was approved by the Research Ethics Committee of the Hospital District. Permission to carry out the study was obtained from the relevant organizations. The participants received information both in written and verbal forms and every participant signed a letter of informed consent [26]. The CDs, memory-cards and written interviews were stored in a locked cabinet and could be accessed only by the first author (M.L). The electronic versions of the interviews were saved on a computer safeguarded by a password.

### 2.2. Participants

The participants were persons who had used mental health and/or substance abuse services or were using these services during the time of the study. A total of 27 service users participated in the study. When selecting the participants, the purpose was to maximize the variation in description of the phenomenon (SUI). According to Patton [27], the strength of this kind of purposeful sampling lies in selecting information-rich cases. There are several different strategies for purposefully selecting the participants. In this study, maximum variation, snowball, and criterion sampling were applied. 

In the study, maximal variation was applied when including service users with experience of mental health and/or substance abuse services, inpatient and/or outpatient services, service users of both sexes and of different ages, from urban and rural areas. Ten women and 17 men participated in this study. During the interviews, the participants were not obliged to talk about their background or medical history but the majority of them did talk to the interviewer about their background (incl. age), illness, diagnosis, and their history of using services. Five participants were under the age of 30 years, nine were between 31 and 50 years and four participants were older than 50 years. As described by the participants themselves, twelve of them had only used mental health services, four only substance abuse services, and eleven had used both mental health and substance abuse services. 

Snowball sampling was used to get in contact with persons, who were no longer actively using mental health or substance abuse services. We contacted service user organizations and peer groups for participants. After one member of a group or organization had participated in the study, he or she could recommend another participant. Criterion sampling was applied especially when recruiting the participants in inpatient care. The selection criteria were age (18–65 years) and the patient's ability to give informed consent evaluated by nursing staff. 

### 2.3. Data Collection

When applying a phenomenographic approach, the study material is usually collected by means of interviews [17]. In this study, semistructured interviews conducted by the first author were used. Service users in out-patient units were contacted through personnel. A brochure of the study was given to the clients, and the persons interested in participating then contacted the interviewer. To get in contact with the users of services in in-patient care, the personnel of these units was consulted first. The personnel then evaluated the patients' ability to give informed consent and to participate in the study. Those service users willing and able to participate received a brochure about the study and contacted the interviewer, with the help of the staff, to arrange the interview. Before these interviews, participants were told that participation was voluntary and participating had no effect on their care and treatment, that anonymity was guaranteed, and that they were able to withdraw from the study at any point. The interviews with the service users in in-patient care took place at the ward premises. Otherwise the participants' interviews took place at their homes or in other place reserved for that purpose. 

The interview themes were selected beforehand and special attention was paid to the entry question, while the subsequent dialogue proceeded according to the participant's answers [28]. The themes and the questions of each interview dealt with the participant's conceptions of SUI in mental health and substance abuse work. The first question in the interviews was “In your opinion, what does service user involvement in mental health and/or substance abuse services mean?” During the interviews, some participants found it difficult to answer the entry question, so additional questions such as “Have you had an opportunity to participate in your own care?” or “In what ways have you participated in your care?” were asked to clarify the concept of user involvement. 

The interviews were audio-taped excluding four cases; in one case the recorder did not work, and in three cases the participant did not give consent for recording. In these cases, notes were made during the interview and complemented afterwards. The audio-taped interviews were transcribed verbatim.

### 2.4. Data Analysis

The purpose of data analysis is to find and define meanings expressed in the interviews, the meanings are then grouped into categories describing the data and the conceptions participants hold of the phenomenon of interest [18, 20]. In this study, the analysis of the participants' conceptions of SUI in mental health and substance abuse work was carried out in three phases by the first author (Table 1).

1. The interviews were transcribed verbatim and the material was compared with the tapes. Every interview was then listened to and read several times in order to get an overall impression and familiarity with the material. Meaningful and adequate expressions related to the study questions were searched for and identified from the material. These expressions were formed into meaning units.

(2) In the second phase, the meaning units were compared with each other with a focus on similarities and differences. Groups of meaning units were formed. The meaning units were also compared repeatedly with the original material in order to ensure the accuracy of the interpretations made. It is important that the researcher recognizes his or her own preconceptions and experiences and is able to bracket them [22].

(3) In the third phase, the groups of meaning units were unified as categories of description. The content of the categories was compared within and between the categories of description. The categories were named to emphasize their content [26]. The categories of description are presented horizontally because they are of equal value. If a category of description would consist of subcategories, they would be vertically related to it and specify it [19, 20].

## 3. Results

**Figure 2:**
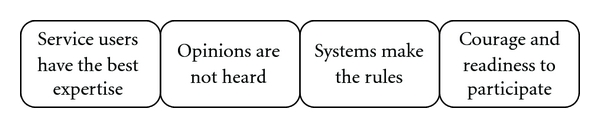
The conceptions of participants concerning SUI in mental health and substance abuse services.

As a result of the data analysis, four qualitatively different categories describing the participants' conceptions of SUI in mental health and substance abuse work emerged: service users have the best expertise, opinions are not heard, systems make the rules, and courage and readiness to participate (Figure 2). The participants talked about service user involvement in personal care and treatment and in the development and delivery of mental health and substance abuse services. Excerpts from the original interviews are included in the description of the categories to convince the reader of the validity of the categories [19]. The letters before the quotations have the following meanings: S: service user, I: interviewer.

### 3.1. Service Users Have the Best Expertise

According to the participants, involving service users in the planning and development of mental health and substance abuse work was necessary in order to achieve change. Some of the participants thought that planning without service users would be useless. If plans were made without experts through experience, all you would achieve would be paper. To know what to develop and in which direction is difficult without personal knowledge of mental health or substance-related problems. In the interviewees' opinion, persons with personal knowledge and experience had the best expertise concerning the content of mental health and substance abuse services.

*Example  1*. S: Well, you can collect fees from the meetings and spend ten million euros, and all you get is zero. All you get is a pile of paper collecting dust in the archives, that's all. Sure, it's important that professionals think about these things, but those who have to deal with them should be involved. This is a kind of disease you can only comprehend if you have experience of your own. Nobody else understands what it means.

*Example  2*. S: Well, it (involving users) could not do any harm, could not it? They know better than the staff. How would I put it? If you have been a patient yourself, you know better what's been done to you and what has not. Nurses do not necessarily know that. Some of them do, but they have not been patients themselves.

### 3.2. Opinions Are Not Heard

Even though service users have useful and valuable expertise, the participants had the conception that SUI did not always happen in practice. The opinions and experiences of service users were ignored. Listening and valuing service users' expertise required time and giving up paternalistic thoughts about service users. According to the participants, service users in mental health and substance abuse services still encountered negative attitudes and bias. Their ability to be involved, to participate and express their opinions was questioned. SUI entailed a division of power; those in power were not willing to share it.

*Example  3. *S: The decision-makers want to decide. Why should they ask service users' opinions. They want to make the decisions for us. It's easier to make decisions when you do not know the problem, or the heart or the core of the problem. Maybe it would be more difficult to make decisions if those concerned had their voice heard. You could see how difficult and complex the problems really are.

*Example  4. *I: You said that service users' opinions are not often asked for. In your opinion, why is it so? S: These kinds of (involving) activities are just evolving, but they are becoming more and more common, and more and more often people are asking users' opinions. It was only a little time ago when patients were kept in institutions. Everything is evolving bit by bit. Even persons recovering from mental health problems have many kinds of experiences and things to say. And in the end, they (services) should work on the patients' conditions as much as possible. The kind of top down dictating is over.

### 3.3. Systems Make the Rules

The mental health and substance abuse service system and organizational culture limited the achievement of SUI. Many laws and acts regulated mental health and substance abuse work; the organizations were often hierarchical and inflexible. Certain regulations existed in outpatient and inpatient care, and a service user had to adapt to those rules. According to the participants' concepts, especially in inpatient care, there were many rules to obey. The meaning or the purpose of those regulations was not always clear to service users.

*Example  5. *I: Have you been able to participate in your own care, for example? S: Well, a little. You can of course ask for a referral to another ward and so on. But the time you stay in the hospital and things like that, they are decided mostly by the doctor. I have noticed that a doctor might have a certain style; for example, in one ward the time spend in the hospital was three weeks no matter what your diagnosis or condition was.

*Example  6. *S: To be able to influence something, that's something that does not fit here in hospital. Is not influencing something the politicians do? If you are here in compulsory treatment, it's another thing. Yes, here you have certain rules and they won't be changed because of one patient, and these things you just have to accept. And nothing to it.

### 3.4. Courage and Readiness to Participate

The courage of users of services and their readiness for involvement and participation varied. Mental health and substance abuse problems still leaded to stigma, bias, and prejudices. Because of that, some service users did not want to take part, for example, in peer groups. A service user's own mental and physical condition, medication and recovery affected their ability and readiness to participate. In Finland, people are reserved, and active involvement and participation or public expressions of opinions are not common. This same cultural feature could also be seen concerning SUI. Some of the participants thought that there was no need to involve service users. In their opinion, it was better to turn to professionals and trust them.

*Example  7. *S: Well, every case is unique. But if you are on medication, if you have many drugs, you do not have the strength to participate because you have enough problems of your own. But then there are those who only have light medication. These persons should be noticed. In my opinion, they are willing to participate and work as peers. And there are many of these kinds of persons. Yes, I'd say 50 percent of all service users would be willing to participate. They see it as part of their recovery.

*Example  8. *Well, they (staff) have listened to my opinions. This is such a difficult disease. These voices when they get loud…The doctors know. They know what it's about. I cannot do much to help myself.

## 4. Discussion

### 4.1. Methodological Aspects

Methodologically, it can be argued that the phenomenographic approach is well suited to research concerned with different conceptions people hold of diverse phenomena. When applying an inductive approach, it was possible to form the categories of description from the rich and extensive data. This study confirmed that service users' opinions are worth listening to, and that they have experiential knowledge to be used in the development of mental health and substance abuse work. Even severely ill persons and patients in inpatient care can participate in studies if data collection methods are appropriate. 

The phenomenographic approach does not attempt to achieve an absolute truth, such a truth does not exist. Appropriate, acceptable and defendable interpretations are the goal [19]. The credibility of the study is discussed in the light of concepts introduced by Fridlund [29] and Fridlund and Hildingh [30]. There are four concepts that are of general importance for scrutinizing a study: applicability, concordance, security, and accuracy. When applying a qualitative method other concepts such as identification, reasonableness, trustworthiness, and conscientiousness should be used. 

To ensure *identification;* the data was acquired by means of interviews and the participants were selected by purposeful sampling [27]. The aim was maximal variation, that is, to get as many qualitatively different conceptions of SUI as possible. *Reasonableness* refers to the validity of the study. In this study, the theoretical framework and research questions guided the formulation of the interview themes. Pretesting of the themes might have improved the reasonableness of the study. *Trustworthiness* is connected with the reliability of the study [30]. The accurate and detailed description of the data analysis and the direct quotations illuminating the categories of description add to the trustworthiness of this study [28]. Trustworthiness is also strengthened by the fact that the first author conducted all the interviews. *Conscientiousness* means that the researcher is aware of his or her own preconceptions and experiences throughout the research process [30]. In this study, the data was read and reflected on repeatedly, and the meaning units and categories were continuously compared to the original data in order to ensure the accuracy of the interpretations made.

### 4.2. Discussion of the Results

This study provided new information about SUI in mental health and substance abuse work from the service user's viewpoint. Methodologically, the phenomenographic approach enabled the service users' involvement and participation in this study. With an inductive approach, their conceptions were listened to and heard. Mental-health and substance-abuse-related problems are a significant challenge to both public health and finances. In the EU, for example, about 11 percent of the population (almost 50 million citizens) are estimated to encounter mental health problems, depression being the leading health problem in many EU countries [31]. In Europe, promoting mental health, reducing stigma, discrimination, and social exclusion, and preventing mental health problems are priorities for the next ten years [32]. On a practical level, involving service users in planning, development and service delivery can support the addressing of these challenges within mental health and substance abuse work. The results of this study can be utilized to understand the essence of SUI, to analyze the barriers to its achievement, and to work towards its implementation. Thus, these results can contribute to improving the education, practice, and management of mental health and substance abuse work.

#### 4.2.1. The Expertise of the Service Users

According to the results, service users have deep, experiential knowledge that should be used in individual care planning, as well as in the development, evaluation, and organizing of mental health and substance abuse services. The need to utilize this expertise has also been highlighted in previous studies [33–35]. SUI is particularly important in the planning, implementation, and management of services [34, 36]. The results of this study confirmed that the achievement of SUI in mental health and substance abuse work is still insufficient. This gap between the rhetoric of involvement and the reality in mental health and substance abuse practice has also been discovered in other studies [3, 4, 34, 37].

#### 4.2.2. Obstacles to SUI

Not all service users want to participate. The state of mental and physical well-being, medication, and personal recovery all affect the service user's capability to be involved, as also argued in previous studies [5, 38, 39]. Some service users are not willing or motivated to participate; they would rather trust in professionals and their decisions. Bryant et al. [38] found in their study that not all users of drug treatment services felt the need to be involved, but rather wanted to concentrate on their own care. Also, a lack of interest and general apathy [5] and passivity [40] may affect the readiness for involvement and participation. 

Hence a challenge for SUI is how to involve users who are passive and not interested in involvement and participation [41]. In order to encourage diverse service users to participate, different, flexible, and innovative forms of involvement are needed [13, 42]. According to the results, organizational culture and negative attitudes can hinder involvement. On the other hand, structures and organizational culture may facilitate SUI [43]. An organization's commitment to SUI [4], and continuing support [43, 44] can promote the staff's commitment to involvement. 

The users of mental health and substance abuse services still encounter negative attitudes and prejudices. In previous studies, the importance of attitudes has also been highlighted [9, 36, 40, 45]. Special attention should be paid to the paternalist or negative attitudes of the staff [4, 38]. Staff education is one way to promote SUI [36, 46]. The participants described conceptions of how the stigma related to mental health and substance abuse problems hindered involvement and participation. The results of this study support the views presented in earlier studies [47–50] indicating that stigma and self-stigma are related to mental health and substance abuse problems.

#### 4.2.3. Lack of Information

This study revealed that service users did not always know the meaning or purpose of various rules and decisions. Services users need adequate and comprehensible information in order to be able to participate and to involve themselves. Sufficient information promotes service users' possibilities to participate in their individual care [5, 38, 51, 52]. Diverse methods of patient education need to be developed [53]. Information sharing alone is not enough; information should be available repeatedly and in a form that service users can understand [40, 54].

#### 4.2.4. New Expertise

SUI changes perceptions of professional expertise. Tritter and McCallum [13] point out that service users may emphasize different questions and issues than professionals. SUI requires power-sharing and a new kind of expertise on the part of professionals. In this study, the participants reported that service users' opinions are not always heard or taken into account. Poulton [54] stresses professionals' need to get away from professional protectionism and medical paternalism to be able to share information and power with service users. As long as SUI remains in control of services providers, it will reinforce the dominant division of power and knowledge [55]. New models and approaches of involvement need to be developed. 

## 5. Conclusions

Service users conceptualize SUI in mental health and substance abuse services from four different perspectives. Service users have deep, experiential knowledge that should be used in individual care planning, as well as in the development, evaluation and organizing of mental health and substance abuse services. The achievement of SUI in mental health and substance abuse work is still insufficient. There are several obstacles to SUI, and different forms of involvement are needed for different kinds of service users to be involved. Special attention should be paid to the provision of adequate information and genuine opportunities to participate. SUI challenges professionals to share power and to develop a new kind of expertise. Within mental health and substance abuse services, nurses often work in close contact with service users. Hence they have excellent opportunities to promote involvement and to encourage service users to get involved and participate.

##  Conflict of Interests

The authors declare that they have no competing interests.

## Figures and Tables

**Table 1:** An example of the data analysis concerning the category of description “Courage and readiness to participate”.

Original interview	Meaningful expressions	Meaning units	Category of description
“Well, every case is unique. *But if you are on medication, if you have many drugs, you do not have the strength to participate* because you have enough problems of your own. But then *there are those who only have light medication. These persons should be noticed. In my opinion, they are willing to participate and work as peers*. And there are many of these kinds of persons. Yes, I'd say 50 percent of all service users would be willing to participate. They see it *as part of their recovery.” *	*if you are on medication, if you have many drugs, you do not have the strength to participate*	Effects of medication	
		Strength to participate	Courage and readiness to participate
	*there are those who only have light medication. These persons should be noticed. In my opinion, they are willing to participate and work as peers*	Willingness to participate	
	*as part of their recovery.” *	Involvement as part of recovery	
